# Progressive decay of Ca^2+^ homeostasis in the development of diabetic cardiomyopathy

**DOI:** 10.1186/1475-2840-13-75

**Published:** 2014-04-09

**Authors:** Shu-Mei Zhao, Yong-Liang Wang, Chun-Yan Guo, Jin-Ling Chen, Yong-Quan Wu

**Affiliations:** 1 Cardiovascular Center, Beijing Friendship Hospital, Capital Medical University, 95 Yong’an Road, XiCheng District, Beijing, China

**Keywords:** Spontaneous Ca^2+^ spark, Cardiac dysfunction, Calcium-associated protein, Diabetic cardiomyopathy

## Abstract

**Background:**

Cardiac dysfunction in diabetic cardiomyopathy may be associated with abnormal Ca^2+^ homeostasis. This study investigated the effects of alterations in Ca^2+^ homeostasis and sarcoplasmic reticulum Ca^2+^-associated proteins on cardiac function in the development of diabetic cardiomyopathy.

**Methods:**

Sprague–Dawley rats were divided into 4 groups (n = 12, each): a control group, and streptozotocin-induced rat models of diabetes groups, examined after 4, 8, or 12 weeks. Evaluations on cardiac structure and function were performed by echocardiography and hemodynamic examinations, respectively. Cardiomyocytes were isolated and spontaneous Ca^2+^ spark images were formed by introducing fluorescent dye Fluo-4 and obtained with confocal scanning microscopy. Expressions of Ca^2+^-associated proteins were assessed by Western blotting.

**Results:**

Echocardiography and hemodynamic measurements revealed that cardiac dysfunction is associated with the progression of diabetes, which also correlated with a gradual but significant decline in Ca^2+^ spark frequency (in the 4-, 8- and 12-week diabetic groups). However, Ca^2+^ spark decay time constants increased significantly, relative to the control group. Expressions of ryanodine receptor 2 (RyR2), sarcoplasmic reticulum Ca^2+^-2ATPase (SERCA) and Na^+^/Ca^2+^ exchanger (NCX1) were decreased, together with quantitative alterations in Ca^2+^regulatory proteins, FKBP12.6 and phospholamban progressively and respectively in the diabetic rats.

**Conclusions:**

Ca^2+^ sparks exhibited a time-dependent decay with progression of diabetic cardiomyopathy, which may partly contribute to cardiac dysfunction. This abnormality may be attributable to alterations in the expressions of some Ca^2+^-associated proteins.

## Background

Ca^2+^-induced Ca^2+^ release plays an important role in the translation of electrical signals to physical contraction in cardiomyocytes, known as excitation-contraction coupling. A small amount of Ca^2+^ influx through L-type Ca^2+^ channels can activate a further discharge of Ca^2+^ from the sarcoplasmic reticulum (SR) via ryanodine receptor 2 (RyR2), resulting in an increase in global intracellular Ca^2+^ which activates contraction [1]. Elementary Ca^2+^ release events occur spontaneously as clusters, termed Ca^2+^ sparks [2]. During diastole, myocardial relaxation results from the reuptake of Ca^2+^ into the SR via Ca^2+^-ATPase (SERCA) on the SR, and concurrently Ca^2+^ is pumped out of the cardiomyocytes via the Na^+^/Ca^2+^ exchanger (NCX). An imbalance of Ca^2+^ flux may induce pathological conditions such as arrhythmia and decreased contractility of the cardiomyocytes. It has been reported that a certain portion of the contractile deficit in heart failure is due to an impairment of Ca^2+^ homeostasis [3,4].

Diabetic cardiomyopathy was initially recognized by Rubler et al. [5] in diabetic patients, who exhibited defects in myocardial contraction and relaxation, and increased morbidity and mortality. Cardiac dysfunction in diabetic models may result from a variety of metabolic and biochemical abnormalities, including abnormal Ca^2+^ homeostasis. As shown in a previous study, the ability of Ca^2+^ current to trigger SR Ca^2+^ release is decreased significantly in diabetic cardiomyocytes. Spontaneous Ca^2+^ spark frequency and peak amplitude were also both significantly reduced (*P* < 0.05) in diabetic myocytes compared with the control myocytes [6]. In addition, Choi KM et al. [7] found that the SR Ca^2+^ release rate and Ca^2+^ stores were depressed in the cardiomyocytes of type 1 diabetic rats. These changes may account for cardiac dysfunction in diabetic models. However, inconsistent results have been reported. For instance, according to Yaras et al. [8] the maximal amplitude of Ca^2+^ sparks was not significantly changed, but the spontaneous Ca^2+^ spark frequency was elevated in diabetic cardiomyocytes, compared with control cells.

It is obvious that the role of Ca^2+^ involvement in diabetic cardiomyopathy is still not fully known and requires further investigation. As animal models in different diabetic stages have been used in various studies, we hypothesized that the inconsistent results regarding Ca^2+^ homeostasis were due to different severities and stages of diabetes. Therefore, this study investigated the progressive alterations in Ca^2+^ homeostasis and expressions of SR Ca^2+^- associated proteins in the development of diabetes, and the association between Ca^2+^ homeostasis and cardiac dysfunction in diabetic cardiomyopathy.

## Methods

### Animal models

The Beijing Friendship Hospital Animal Care Committee approved all animal handling protocols. The diabetic rat model was induced with a single intraperitoneal injection of streptozotocin (60 mg/kg diluted in 0.1 M citrate buffer, pH = 4.4; Sigma, USA) in male rats (Sprague–Dawley; 200 ± 20 g). Rats in the control group were given an injection of a matched volume of citrate buffer (0.1 M). Subsequently on day three and day five after the injection, random blood glucose concentrations were measured. Only rats with blood glucose levels ≥16.7 mmol/L on both days were defined as diabetic and used in the study.

Four experimental groups (n = 12, each) consisted of the control group (group A) and the diabetic model rats, examined at 4, 8, or 12 weeks after injection (groups B, C and D, respectively). All the rats were housed (three per cage) in a controlled environment at 20 ± 2°C, 30-70% humidity, and a 12:12 h light–dark cycle, and given standard chow and water ad libitum. In accordance with the protocol, all diabetic model rats were euthanized prior to the experiments at the assigned time points. Control rats were euthanized at the twelfth week after an injection with citrate buffer.

### Echocardiographic measurements

Echocardiography was performed to evaluate cardiac structure and function in all animals involved in the study (n = 12, in each group). The rats were weighed and anesthetized with 10% chloral hydrate at 0.3 mL/100 g body weight [9]. Two-dimensional and M-mode echocardiographic measurements were carried out with a VEVO 770 high-resolution *in vivo* imaging system (VisualSonics, Toronto, Canada). The transthoracic echocardiography images were obtained via long- and short-axis views using standard echocardiography techniques [10]. Cardiac structure was principally evaluated by the left ventricular end diastolic and systolic diameters. The left ventricular systolic function was assessed according to ejection fraction and fractional shortening, and the left ventricular diastolic function was determined by Doppler waveforms of mitral inflows, which were obtained from an apical four-chamber. The variables included the peak early diastolic filling velocity (E wave), the peak late diastolic filling velocity (A wave), and the ratio of the peak early-to late-filling velocity (E/A).

### Hemodynamic measurements

To assess the hemodynamic condition, an ultra-miniature catheter connected to a polygraph instrument (BL-420, TaiMeng, ChengDu, China) was inserted into the carotid artery, and then to the left ventricle of the anesthetized animals (n = 6, in each group). After a 5-min period of stabilization, the parameters were acquired and recorded. The myocardial contractility was assessed according to the left ventricular systolic peak pressure (LVPSP) and the maximum rate of ascending pressure change in the left ventricle (+dP/dt_max_). Myocardial relaxation was evaluated according to the left ventricular end-diastolic pressure (LVEDP) and the maximum descending rate of left ventricular pressure (−dP/dt_max_) [11]. The hearts were then removed and stored at −80°C for further study.

### Examination of Ca^2+^ homeostasis in cardiomyocytes

Ventricular myocytes were isolated from the rats (n = 6, in each group) as described previously [12]. In brief, the hearts were cannulated, and perfused for 10 minutes at 2 mL/min (37°C) with oxygenated Ca^2+^-free buffer, containing 145 mM NaCl, 5 mM KCl, 1.2 mM MgSO_4_, 1.4 mM Na_2_HPO_4_, 0.4 mM NaH_2_PO_4_, 5 mM HEPES, and 10 mM glucose; pH 7.4, adjusted with NaOH. Then the hearts were perfused with an enzyme solution, containing 0.6 mg/mL collagenase type II (Invitrogen, USA) for about 15 min. The ventricular muscle tissue was cut into small pieces, and the ventricular myocytes were released by agitating the cell/tissue suspension. After filtration through a nylon mesh, Ca^2+^ was gradually reintroduced up to 1.8 mM in the cell suspension. The ventricular myocytes were then ready to be used in further studies.

For the Ca^2+^ spark imaging, cardiomyocytes were incubated with 10 μM Fluo-4 AM (Invitrogen, USA) in a normal Tyrode’s solution for 5 min at 37°C, and transferred into a recording chamber. Cells were perfused with the Tyrode’s solution for 20 min for de-esterification. SR Ca^2+^ load was estimated by a rapid application of 10 mM caffeine via a nearby pipette. To control the rest potentiation, experiments were performed after about 30 minutes, upon reintroduction of Ca^2+^ to a concentration of 1.8 mM in each group. Confocal line-scan imaging was performed using a Leica SP5 confocal microscope (Germany, 40 ×, 1.25 NA) with an excitation at 488 nm. Line-scan images were acquired at a sampling rate of 1.43 ms per line, along the longitudinal axis of the cell. Digital image processing was performed using MATLAB 7.1 (Math Works). For a minimal detection of Ca^2+^ sparks, the criteria was set at greater than 3.8× the standard deviation (SD) of the background noise over the mean background noise, and Ca^2+^ sparks were automatically counted with the Sparkmaster Plug-in for Image [13].

### Western blot analysis

Western blot analyses (n = 6, in each group) were conducted as previously described [14] to assess the expressions of the Ca^2+^-associated proteins RyR2, SERCA, NCX1, FKBP12.6, and phospholamban (PLB), and the phosphorylation status of PLB at serine-16 (PLB-Ser16) and threonine-17 (PLB-Thr17). In brief, the heart tissue was homogenized in a cold Tris–HCl buffer (120 mM NaCl, 1.0%, Triton X-100, 20 mM Tris–HCl, pH 7.5, 10% glycerol, 2 mM EDTA, protease inhibitor cocktail). Total protein was quantified using a BCA protein assay kit as required (CW Bio Tech, 02912E, China). Equal amounts of protein (40 μg) were separated by SDS-PAGE. After electrophoresis, proteins were transferred to a polyvinylidene fluoride membrane and blocked with Tris buffered saline containing 5% skim milk powder and 0.05% Tween 20. Then the corresponding primary antibodies, RyR2 (1:1000, Abcam, USA), SERCA (1:1000, Abcam, USA), NCX1 (1:300, Santa Cruz, USA), PLB (1:1000, Abcam, USA), PLB-Thr17 (1:300, Santa Cruz, USA), PLB-Ser16 (1:300, Santa Cruz, USA), and FKBP12.6 (1:1000, R&D, USA) were added in series. The membranes were incubated with the diluted antibody preparations overnight at 4°C. After washing, the membranes were incubated with horseradish peroxidase (HRP)-conjugated goat anti-rabbit IgG (H + L) and goat anti-mouse IgG (H + L) antibodies (1:10^4^; Jackson, USA) for 40 min at room temperature. The blots were visualized using an enhanced chemiluminescence detection kit (Millipore, USA). Target proteins were quantified and normalized relative to β-actin (1:1000, Zhongshan, China).

### Statistical analysis

Data were evaluated by one-way ANOVA expressed as the mean ± SD. When a significant difference was identified by ANOVA, a post-hoc analysis was performed using the Student-Newman-Keuls test. Data were analyzed using SPSS 13.0 software, and a *P*-value < 0.05 was considered significant.

## Results

### Descriptive variables of diabetic rats

Blood glucose levels were significantly higher in all diabetic rats (22.62 ± 5.07 mmol/L) compared with the control rats (7.57 ± 1.69 mmol/L; *P =* 0.00), but were not significantly different among the diabetic groups (*P >* 0.05; Table 1). Relative to the body weight of the control rats, that of the streptozotocin-induced diabetic rats was significantly lower, and progressively decreased from 4 weeks (group B) to 12 weeks (group D, *P* < 0.05). Heart weights also decreased during the 12-week development of diabetes, although the difference was statistically significant only in group D compared with the control group (*P* = 0.013).

**Table 1:** General characteristics and results of echocardiography

	**Control**	**Diabetic models**		
	**Group A**	**Group B**	**Group C**	**Group D**
Body weight (g)	589.83 ± 37.4	450.3 ± 52.6^a^	394.8 ± 62.3^a^	256 ± 46.3^a,b,c^
Heart weight (mg)	780 ± 105.2	796.8 ± 104.8	748.5 ± 152.2	595.1 ± 59.6^a,b,c^
Blood glucose (mmol/L)	7.57 ± 1.69	24.83 ± 6.19^a^	22.25 ± 5.16^a^	19.47 ± 2.63^a^
Echocardiography				
Heart rate (beats/min)	420 ± 15	392 ± 41	409 ± 34	388 ± 30
LVEDD (mm)	11.67 ± 2.06	7.7 ± 0.6^a^	7.8 ± 0.5^a^	7.5 ± 0.4^a^
LVESD (mm)	5.84 ± 1.15	3.9 ± 0.3^a^	4.3 ± 0.7^a^	4.1 ± 0.6^a^
Ejection fraction (%)	76.1 ± 4.2	71.4 ± 4.9	71.7 ± 3.8	67.7 ± 5.18^a^
Fractional shortening (%)	45.4 ± 2.9	41.6 ± 3.7	39.9 ± 3.1	37.6 ± 2.8^a^
E wave (cm/s)	129.2 ± 6.74	124.8 ± 6.13	120.9 ± 8.32	112.9 ± 5.93
A wave (cm/s)	76.5 ± 9.1	79.3 ± 10.5	73.1 ± 7.58	79.1 ± 5.39
E/A ratio	1.69 ± 0.2	1.56 ± 0.26	1.65 ± 0.17	1.42 ± 0.14^a^

LVEDD, left ventricle end-diastolic diameter; LVESD, left ventricle end-systolic diameter.

^a^*P* < 0.05 compared with control group; ^b^*P* < 0.05 compared with group B; ^c^*P* < 0.05 compared with group C.

### Echocardiography measurements

**Figure 1:**
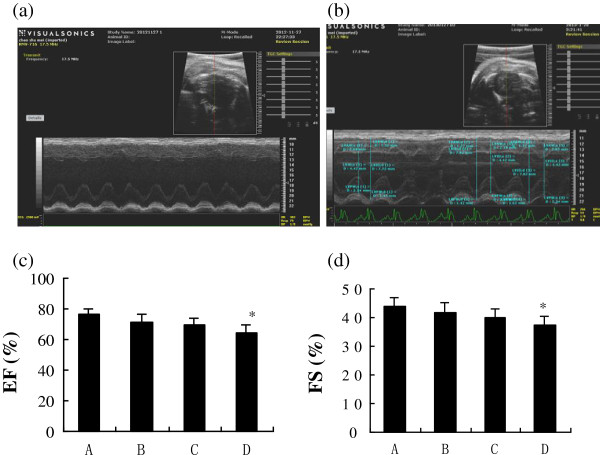
**Echocardiography of the study subjects with ejection fraction and fractional shortening. (a)** Representative echocardiograph. **(b)** Representative measurement of the parameters. **(c)** Ejection fraction (EF) values of groups A-D. **(d)** Fractional shortening (FS) values of groups A-D. *P < 0.05, compared with group A.

Similar heart rates were observed in all groups (*P* = 0.627; Table 1). The left ventricular end-diastolic diameters (LVEDD) and left ventricular end-systolic diameters (LVESD) were comparable in all 3 diabetic groups (*P >* 0.05). With regard to ejection fraction (EF) and fractional shortening (FS), both showed a trend toward gradual decline in the diabetic rats over the 12 weeks, with significant reductions (*P <* 0.05) appearing at the twelfth week after diabetes was induced (Figure 1). E waves exhibited a downward trend during the progression of diabetes, but there were no significant differences among the diabetic groups (*P >* 0.05). In group D, there was a slight but substantial decline in E/A ratio compared with that in control group (*P =* 0.027).

### Hemodynamic measurements

**Figure 2:**
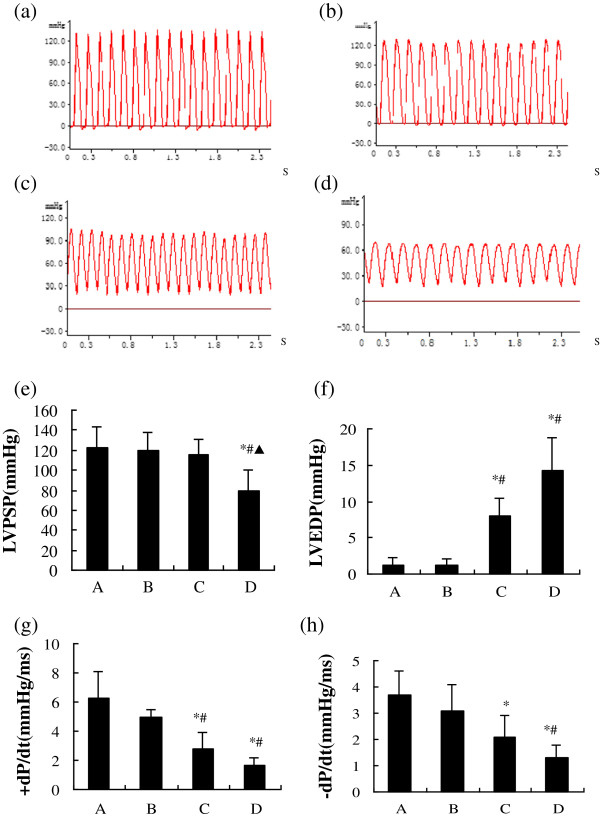
**Cardiac hemodynamics.** The representative hemodynamic curves recorded by polygraphy: **(a)** Group A; **(b)** Group B; **(c)** Group C; **(d)** Group D. X-axes represent time in second. The parameters: left ventricular systolic peak pressure (LVPSP) **(e)**, left ventricular end-diastolic pressure (LVEDP) **(f)**, +dP/dt_max_**(g)**, −dP/dt_max_**(h)** in groups A-D. ±dP/dt_max_: maximal ascending and descending rates of left ventricular pressure. *P < 0.05, compared with group A; ^#^*P* < 0.05, compared with group B; ^▲^*P* < 0.05, compared with group C.

As diabetes progressed over the experimental period, LVPSP decreased gradually from group B (4 weeks) to group D (12 weeks; Figure 2e). Compared with the controls, LVPSP was reduced significantly in group D (*P =* 0.009), which was consistent with the marked drops in EF and FS. Concurrently, the left ventricular end-diastolic pressure (LVEDP) increased gradually. Relative to the control rats, a significant increase in LVEDP first appeared in group C (8 weeks, *P =* 0.003, Figure 2f), which was earlier than that of the significant difference in LVPSP. Both + dP/dt_max_ and –dP/dt_max_ began to decrease significantly in group C, and were sustained in group D, which suggested marked impairments of both systolic and diastolic capabilities of the diabetic myocardium (Figure 2g and h).

### Maintenance of Ca^2+^ homeostasis

**Figure 3:**
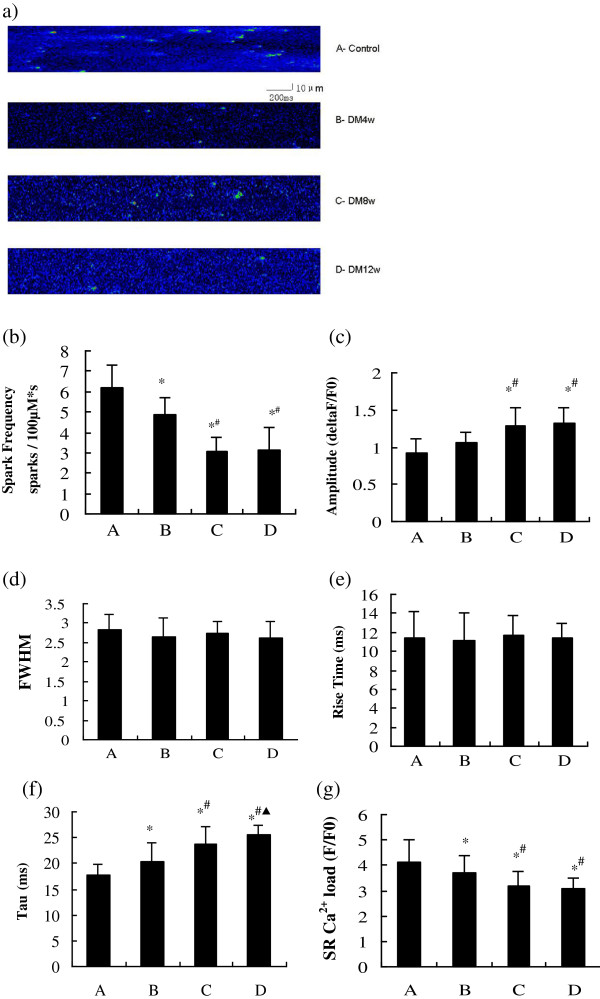
**Ca**^**2+ **^**sparks in groups A-D. (a)** Images of Ca^2+^ sparks; **(b)** Ca^2+^ spark frequency; **(c)** peak amplitude of Ca^2+^ sparks; **(d)** FWHM of Ca^2+^ sparks; **(e)** rise time; **(f)** Tau; **(g)** SR Ca^2+^ load. *P < 0.05, compared with group A; ^#^*P* < 0.05, compared with group B; ^▲^*P* < 0.05, compared with group C.

Over the 12-week progression of diabetes, there were alterations in the properties of Ca^2+^ sparks (Figure 3). The frequency of Ca^2+^ sparks showed a significant decline, beginning at 4 weeks (group B), and was sustained in groups C (8 weeks) and D (12 weeks; Figure 3b). Concurrently, relative to the control group the Ca^2+^ spark peak amplitude (ΔF/F) was unchanged in group B, but significantly increased from group C to group D (Figure 3c). The Ca^2+^ spark decay time constant (Tau) increased significantly and progressively from group B to group D (Figure 3f).

In addition, caffeine stimulation caused a sudden and transient Ca^2+^ release from the SR and an increase in intracellular Ca^2+^ concentration, which represented the SR Ca^2+^ load. The SR Ca^2+^ load showed a marked decline, which was first observed in group B, and declined further in groups C to D (Figure 3g). However, neither the Ca^2+^ spark rise times nor the full width at half-maximum amplitude (FWHM) were significantly different in the different stages of diabetes from that of the control rats (Figure 3d and e).

### Expressions of Ca^2+^-associated proteins

**Figure 4:**
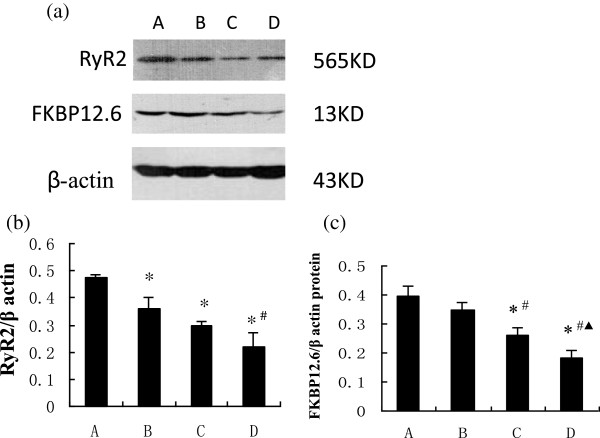
**RyR2 and FKBP12.6 in groups A-D. (a)** Western blots of RyR2, FKBP12.6, and β-actin; **(b)** ratios of RyR2 to β-actin; and **(c)** ratios of FKBP12.6 to β-actin; *P < 0.05, compared with group A; ^#^*P* < 0.05, compared with group B; ^▲^*P* < 0.05, compared with group C.

**Figure 5:**
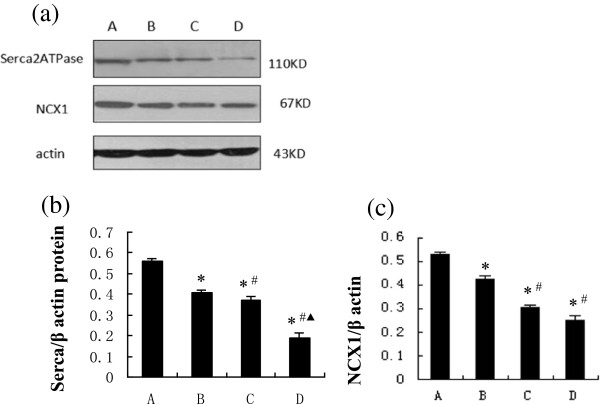
**SERCA and NCX1 in groups A-D. (a)** Western blots of SERCA, NCX1, and β-actin; **(b)** ratios of SERCA to β-actin; and **(c)** ratios of NCX1to β-actin. *P < 0.05, compared with group A; ^#^*P* < 0.05, compared with group B; ^▲^*P* < 0.05, compared with group C.

**Figure 6:**
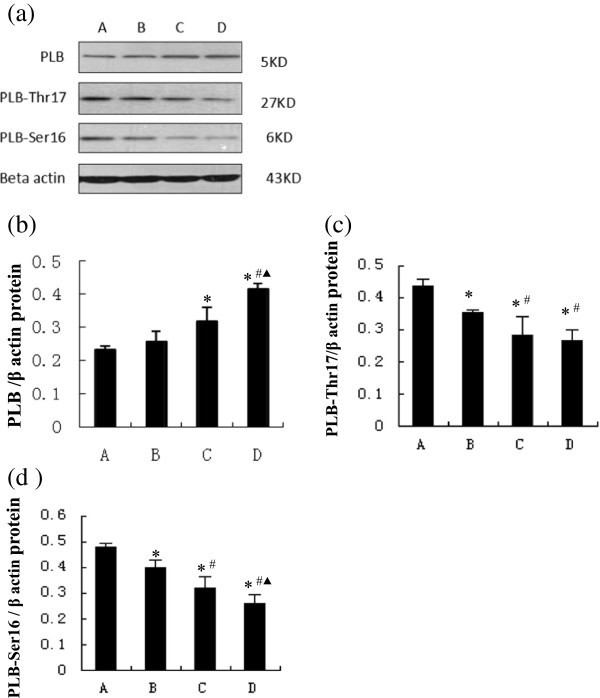
**PLB, PLB-Thr17 and PLB-Ser16 in groups A-D. (a)** Western blots of PLB, PLB-Thr17, PLB-Ser16 and β-actin; Relative ratios of PLB **(b)**, PLB-Thr17 **(c)** and PLB-Ser16 **(d)** to β-actin. *P < 0.05, compared with group A; ^#^*P* < 0.05, compared with group B; ^▲^*P* < 0.05, compared with group C.

RyR2, SERCA, and NCX1 are the major channel proteins for Ca^2+^ flux, and their expressions were analyzed by western blot (Figures 4 and 5). Levels of RyR2 proteins were significantly lower in group B, relative to those of the control, with progressively less in groups C and D (Figure 4b). Expressions of FKBP12.6 protein, a regulatory protein of RyR2, were similar in the control rats and group B, but significantly less in group C, and reduced further in group D (Figure 4c). Both SERCA and NCX1 showed a trend of gradual decline over the 12 weeks in diabetic rats: significantly less in group B relative to the controls, and further declines from group C to group D (Figure 5b, 5c). In addition, expressions of PLB protein, a regulatory protein of SERCA, in group B were similar to those in the control, but significantly and progressively higher levels were detected from group C to group D (Figure 6b). Concurrently, expressions of PLB-Thr17 and PLB-Ser16 proteins were also significantly less in group B relative to the controls, and further declines were detected from group C to group D (Figure 6c, d).

## Discussion

The present study assessed changes in Ca^2+^ homeostasis and sarcoplasmic reticulum Ca^2+^-associated proteins in the development of diabetic cardiomyopathy in a rat model. Over the 12-week experimental period, the cardiac function of the diabetic rats gradually worsened, and this was positively associated with changes in Ca^2+^ homeostasis. As diabetes progressed, Ca^2+^ spark properties changed and levels of RyR2, SERCA, NCX1, and FKBP12.6 decreased.

With the development of diabetes, cardiac function of the rats gradually declined. Echocardiography showed impairments of systolic and diastolic functions, reflected by marked reductions in EF, FS, and E/A ratio, which concurrently appeared at the twelfth week.

Hemodynamic measurements are more sensitive than echocardiography in the detection of early cardiac dysfunction. The results of hemodynamic measurements in this study indicated that diastolic and systolic myocardial performance was depressed at the eighth week of diabetes, as determined by significant reductions in ± dP/dt_max_. Furthermore, a significant increase in LVEDP appeared at the eighth week of diabetes, earlier than the significant decrease in LVPSP at 12 weeks. These findings suggest that cardiac dysfunction in diabetic rats initially started with left ventricular diastolic function and then proceeded to systolic function, in agreement with previous studies [15]. Furthermore, we noticed that cardiac dysfunction correlated with changes in Ca^2+^ homeostasis over the 12 weeks.

Our data showed that Ca^2+^ spark properties experienced a series of changes in a time-dependent manner, associated with the duration of diabetes. The major changes included a gradual decline in both the Ca^2+^ spark frequency and SR Ca^2+^ load, and simultaneously an increase in both the Tau and peak amplitude of Ca^2+^ sparks. Ca^2+^ spark peak amplitudes and the FWHM reflected the shapes and sizes of Ca^2+^ sparks in the different diabetic periods. A decrease in Ca^2+^ spark frequency could indicate a decline in both SR Ca^2+^ release and global intracellular Ca^2+^ concentration during excitation-contraction coupling, which might account for, at least in part, the gradual impairment of systolic function in the diabetic rats. An increase in Tau (i.e., the Ca^2+^ spark decay time constant) could indicate a decline of Ca^2+^ efflux rate, which might partly account for the reduction of –dP/dt_max_, as well as the impairment of diastolic function in the diabetic rats. The SR Ca^2+^ load gradually declined over the experimental period, and might be partly responsible for the drop in Ca^2+^ spark frequency in the diabetic rats. It was also noticed that cardiac dysfunction occurred after alterations in Ca^2+^ handling. This implies that the depression of cardiac function may result, at least in part, from progressive Ca^2+^ mishandling.

We also showed that levels of the major Ca^2+^ channel proteins, RyR2, SERCA, and NCX1 declined in a time-dependent manner with the progression of diabetes. Since these declines appeared earlier than Ca^2+^ mishandling, Ca^2+^ mishandling could partly result from the changes in channel protein levels. Those of RyR2, the major Ca^2+^ release channel protein on the SR, exhibited a downward trend in the diabetic rats, which was primarily responsible for the decrease in Ca^2+^ diffusion away from the SR and Ca^2+^ spark decay.

Simultaneously, the levels of FKBP12.6, the regulatory protein of RyR2, also declined in a time-dependent manner in the diabetic rats. FKBP12.6 is a small cytosolic protein which binds to the RyR2 protein. The binding stabilizes the RyR2 in the channel’s closed state during diastole [16]. A reduction of FKBP12.6 protein impairs the binding and increases the open state of the RyR2 during diastole, thereby leading to an aberrant increase in diastolic Ca^2+^ leak [16]. Diastolic Ca^2+^ leak results in a reduction of SR Ca^2+^ load and Ca^2+^ spark frequency, and an increase in the Tau, thereby contributing to the impairment of left ventricular systolic and diastolic functions.

SERCA is another major Ca^2+^ channel protein on the SR, and is responsible for Ca^2+^ reuptake (70-92%) into the SR [17]. NCX1 is the major Ca^2+^ channel protein on sarcolemma, and is responsible for Ca^2+^ efflux from cardiomyocyte during diastole. Reuptake or efflux of Ca^2+^ via SERCA or NCX1 decreases the Ca^2+^ concentration in cardiomyocytes, which facilitates myocardial relaxation and maintains the Ca^2+^ content in the SR. Decreases in SERCA and NCX1 in this study were primarily the result of intracellular accumulation of Ca^2+^ during diastole and a prolonged Tau, which corresponded to the impairment of left ventricular diastolic function in the development of diabetes. Moreover, the reduction in SERCA protein was also related to a decrease in the Ca^2+^ reuptake rate, leading to a decline of SR Ca^2+^ load.

The regulation of SERCA is dependent on interaction with PLB. It has been confirmed that PLB acts as an inhibitor on SERCA, whereas phosphorylation of PLB can attenuate the inhibition and induce a substantial increase in Ca^2+^ flux via SERCA [18-20]. Upon binding with PLB, SERCA loses activity as Ca^2+^ reuptake decreases. In the present study, the PLB protein levels progressively increased over the experimental period, and phosphorylation of PLB experienced a gradual decline in the diabetic rats. Both these changes in PLB and its phosphorylation status attenuated the Ca^2+^ flux by SERCA during diastole in diabetic rats, which was also related to the impairment of left ventricular diastolic function.

## Conclusions

The present study showed that Ca^2+^ sparks decay with progression of diabetes, accompanied by a successive impairment of cardiac function. Alterations in Ca^2+^ -associated protein levels were detected in the development of diabetes, which partly accounted for the Ca^2+^ spark decay and subsequent cardiac dysfunction. These results may indicate a possible molecular mechanism underlying cardiac dysfunction in the diabetic rat model, and thereby contribute to the search for a possible intervention. Further studies are warranted to investigate the signal pathways responsible for alterations in the levels of Ca^2+^-associated proteins.

## Abbreviations

EF: Ejection fraction; FS: Fractional shortening; FWHM: Full width at half-maximum amplitude; LVEDD: Left ventricular end-diastolic diameters; LVEDP: Left ventricular end-diastolic pressure; LVESD: Left ventricular end systolic diameters; LVPSP: Left ventricular systolic peak pressure; NCX: Na^+^/Ca^2+^ exchanger; PLB: Phospholamban; RyR2: Ryanodine receptor 2; SERCA: Sarcoplasmic reticulum Ca^2+^-2ATPase; SR: Sarcoplasmic reticulum; Tau: Ca^2+^ spark decay time constant.

## Competing interests

The authors declare that they have no competing interests.

## Authors’ contributions

S-MZ carried out the examination of Ca^2+^ homeostasis in cardiomyocytes, participated in the sequence alignment, and drafted the manuscript. Y-LW carried out the Western blot analysis. C-YG performed echocardiographic and hemodynamic measurements. J-LC carried out the animal feeding and performed the statistical analysis. Y-QW conceived of the study, and participated in its design and coordination and helped to draft the manuscript. All authors read and approved the final manuscript.
